# Real-Time Straight-Line Detection for XGA-Size Videos by Hough Transform with Parallelized Voting Procedures

**DOI:** 10.3390/s17020270

**Published:** 2017-01-30

**Authors:** Jungang Guan, Fengwei An, Xiangyu Zhang, Lei Chen, Hans Jürgen Mattausch

**Affiliations:** Hiroshima University, 1-3-1 Higashi-Hiroshima, Hiroshima 739-8530, Japan

**Keywords:** Hough Transform, 1-dimensional Hough space, synchronized initialization, video-based straight lines detection

## Abstract

The Hough Transform (HT) is a method for extracting straight lines from an edge image. The main limitations of the HT for usage in actual applications are computation time and storage requirements. This paper reports a hardware architecture for HT implementation on a Field Programmable Gate Array (FPGA) with parallelized voting procedure. The 2-dimensional accumulator array, namely the Hough space in parametric form (*ρ*, *θ*), for computing the strength of each line by a voting mechanism is mapped on a 1-dimensional array with regular increments of *θ*. Then, this Hough space is divided into a number of parallel parts. The computation of (*ρ*, *θ*) for the edge pixels and the voting procedure for straight-line determination are therefore executable in parallel. In addition, a synchronized initialization for the Hough space further increases the speed of straight-line detection, so that XGA video processing becomes possible. The designed prototype system has been synthesized on a DE4 platform with a Stratix-IV FPGA device. In the application of road-lane detection, the average processing speed of this HT implementation is 5.4 ms per XGA-frame at 200 MHz working frequency.

## 1. Introduction

Hough first introduced the Hough Transform (HT) in 1962 [1], and used it to seek bubble tracks rather than extract shapes in images. Duda and Hart then first employed the HT in 1972 [2] to find straight lines in images. Dana H. Ballard used the HT to identify the position of arbitrary shapes in 1987 [3].

Straight-line detection is an important objective in image processing and computer vision. It has been widely used in many industrial applications such as lane detection, unmanned vehicle guidance, robot navigation, medical image processing, computer vision and artificial intelligence [4,5,6,7]. The HT has good stability for the purpose of straight-line detection, as it is robust against many problems like line gaps or noise in real-world applications.

The HT can be viewed as an evidence-gathering approach in an accumulator array followed by a final voting process on this evidence. It defines a mapping process from the Cartesian coordinate space to the polar coordinate Hough space (represented by the accumulator array) by a function, which describes the targeted shape. However, due to high computational complexity and large memory usage, a software implementation for the HT based on general purpose CPUs is not suitable for real-time applications.

On the other hand, hardware implementations for highly complex algorithms can improve the computational performance, by exploiting parallelism, which is a well-known popular approach for achieving real-time implementations. In the literature, the Coordinate Rotational Digital Computer (CORDIC) algorithm [8,9,10,11,12] is often used in hardware implementations for the HT, when no hardware multiplier is available. CORDIC is a simple and efficient algorithm to calculate trigonometric functions, as well as, can transform rectangular to polar coordinates. The CORDIC-based HT increments the angle of the coordinate of a potential line to find all corresponding distance parameters. For each computation of given angles *θ*, to find related radii *ρ*, a series of convergent micro-rotations is executed [9]. It is worthwhile pointing out that accuracy losses of CORDIC depend not only on the number of fractional bits but also on the number of iterations. As one of the best hardware platforms, Field Programmable Gate Arrays (FPGAs) were used for HT implementation in [8,13]. However, insufficient processing speed is still a challenge for implementation of straight-line detection by Hough transform in real-time applications.

Fortunately, the research efforts to improve the HT towards better suitability for real-time applications have intensified. In recent years, many improvements or optimization algorithms were proposed, which focus on the operational process and the voting procedure of HT. A survey of the HT method [14] summarizes these HT-variants and modifications made to overcome the existing limitations. Major variants of HT for straight-line detection include the Probabilistic Hough Transform (PHT), Randomized Hough Transform (RHT) and Digital Hough Transform (DHT) approaches. Both PHT and RHT aim at increasing the operation speed of HT by the way of randomly selecting certain portions (choosing a subset) of the object points (edge pixels) to approximate the complete Hough transform with a small as possible error for extraction of straight lines more quickly. However, in the PHT algorithm, one object point maps into many parameter values (*ρ*, *θ*) (one-to-many mapping), while RHT uses many object points to define a straight line (many-to-one mapping) [15,16]. Although these proposed alternative algorithms could reduce resource consumption and processing time, they do not consider the location errors (errors between the actual line coordinates and digital image coordinates) [17], so that the accuracy is compromised. In order to solve this problem, DHT adopts a pattern of judging whether a given set of object points has the digital straight-line (DSL) property [18]. The DSL property is determined by a deviation value between the actual line coordinates and the digital image coordinates, which means that when the deviation value is less than a fixed threshold the object points are considered to have the DSL property. This variant of HT can significantly improve the accuracy when processing low-resolution images, but it does not have an advantage for dealing with high-resolution images or noisy images. To increase speed, many researchers investigated other algorithms or solutions in combination with HT, such as the combination with neural networks. M. W. Spratling et al. [19] proposed a new method for implementing the voting process of the HT, which employs a competitive neural-network algorithm to perform a form of probabilistic inference known as “explaining away”. This method could achieve straight-line identification that is more accurate when compared with the standard voting process of the HT. In [20], the authors reported similar improvement characteristics for the modified Hough transform (MHT) and the windowed random Hough transform (WHT), employing the “many-to-one” mapping and a sliding window neighborhood technique to alleviate the computational and the storage complexities.

The main limitation of the HT-based line-detection algorithms is the high computational burden. Gradient-based line-detection methods were mainly developed for fulfilling the speed requirements of practical applications. In 1976, O’gorman et al. [21] used the gradient direction to improve the efficiency of the HT, where the accumulator of the voting process is extended with the magnitude of the gradient orientation of each edge pixel. Bonci et al. [22] proposed a method on the basis of an error propagation technique for avoid the noise effects in [21]. In [22], a Bayesian probabilistic scheme is used for efficient consideration of the probability of each edge pixel and calculating the straight-line feature probability. Furthermore, a progressive PHT (PPHT) algorithm was propose in [23] where all edge pixels assigned to the line or added to the accumulator are removed for computing reduction. In recent years, hardware implementations for the gradient-based HT have also been proposed in [24,25,26] for practical applications. In particular, a resource-efficient hardware architecture was proposed to implement the gradient-based HT with image-block level parallelism. Additionally, an off-chip memory was used to store the pre-processed binary-feature image with run-length encoding for reducing the computing complexity.

Video-based line detection is becoming increasingly important in the practical applications. However, the improvements or optimizations of the HT algorithm in the literature still can not completely satisfies the demand for high operation speed in real-time applications. Take lane detection in automotive applications as an example. An important purpose of the lane detection is to warn the driver or an automatic driving system that driving-lane deviation is happening, so that actions can be taken to continue the safe driving and to avoid the occurrence of potential accidents. As the limitations for the necessary response speed of acting appropriately are only 0.4–1.0 s, fast real-time processing of the driving-lane detection is indispensable. Furthermore, the human eye can not distinguish intervals between images when the frame rate is higher than 10–12 frame per second, that is to say the implementation should process more than 10–12 image frames during a time interval of 0.4–1.0 s. Consequently, improving the processing speed of HT implementations remains a key challenge for many practical applications.

The contribution of this paper can be categorized into three parts. First, a look-up table solution for computing sin *θ* and cos *θ* leads to regular increments of *θ* (Δ*θ*), so that the 2-dimensional Hough space can be mapped on a 1-dimensional array. As a result, the computing of *ρ* and voting on a location of (*ρ*, *θ*) can be implemented in a pipeline without address conflicts. Second, the regular increment Δ*θ* and the 1-dimensional array structure allow subdivision of the Hough space into a number of parts with parallelized voting procedure and efficient handling of the computational complexity. Furthermore, the line detection with a thresholding method can be parallelized during the voting procedure without additional processing effort, by using only an additional logic comparator. Third, the concept of parallel initialization for no-longer needed Hough-space parts reduces waiting times between processing of subsequent image frames. Consequently, real-time straight-lines detection for videos with high resolution like XGA becomes possible.

The paper is organized as follows: Section 2 briefly describes the HT algorithm and the common HT criticism. In Section 3, we propose a hardware architecture for HT implementation with pipelined and parallelized architecture for computation and voting procedure. Section 4 evaluates the performance of the developed prototype system on a DE4 FPGA platform. Finally, a conclusion is given in Section 5.

## 2. Hough Transform (HT)

The Hough Transform is a robust and effective method for finding lines in images. It applies the transformation (Equation (1)) from Cartesian (*x*, *y*) to polar (*ρ*, *θ*) coordinates

(1) ρ=x·cosθ+y·sinθ

where *ρ* is the shortest distance from the origin to the straight line, while *θ* is the angle between the *x*-axis and the vector orthogonal to the line. Equation (1) can be viewed as a parametric equation for all possible straight lines through each point ($x_{i}$, $y_{i}$) in Cartesian space and becomes a sinusoidal curve in the polar (*ρ*, *θ*) space. For all points on a straight line in the Cartesian space, the corresponding sinusoidal curves according to Equation (1) pass through the same point in the (*ρ*, *θ*) space.

The Hough transform exploits above properties by using a discretized (*ρ*, *θ*) space, called Hough space, and the following algorithm:

1. Initialized Hough space with zeros.
2. Select coordinates ($x_{i}$, $y_{i}$) from an edge pixel of the processed image [27].
3. Change *θ* by the discretization steps Δ*θ* from 0 to $\theta_{max}$ and calculate *ρ* according to Equation (1). Increase the vote value of the corresponding discretization bin of the Hough space for each *θ*.
4. If this is the last edge pixel of the image, go to step 1 and the next image. Otherwise, go to step 2.

The common criticism of the Hough Transform is the high computational burden. The discretization parameters Δ*ρ* and Δ*θ* are the main factors determining the size of the Hough space. For each edge point of an image with the coordinate (*x*, *y*), we can obtain the values of *ρ* by incrementing the value *θ* from $0^{\circ }$ to *π* according to Equation (1). The discretization parameters Δ*ρ* and Δ*θ* directly affect the size of the accumulator-array for the Hough space and the detection accuracy for lines.

Note that the computational cost of Hough Transform depends on the number of edge pixels (*p*) and the incremental quantity Δ*θ* of *θ* in the parameter space, so that the computational cost is given by $O(p\cdot \frac{\theta_{max}}{\Delta \theta })$. Hence, a high resolution causes high computational requirements and large memory usage but has better line-detection accuracy.

## 3. Hardware Architecture for HT Implementation

### 3.1. Pipelined and Parallelized ρ and θ Computation

**Figure 1:**
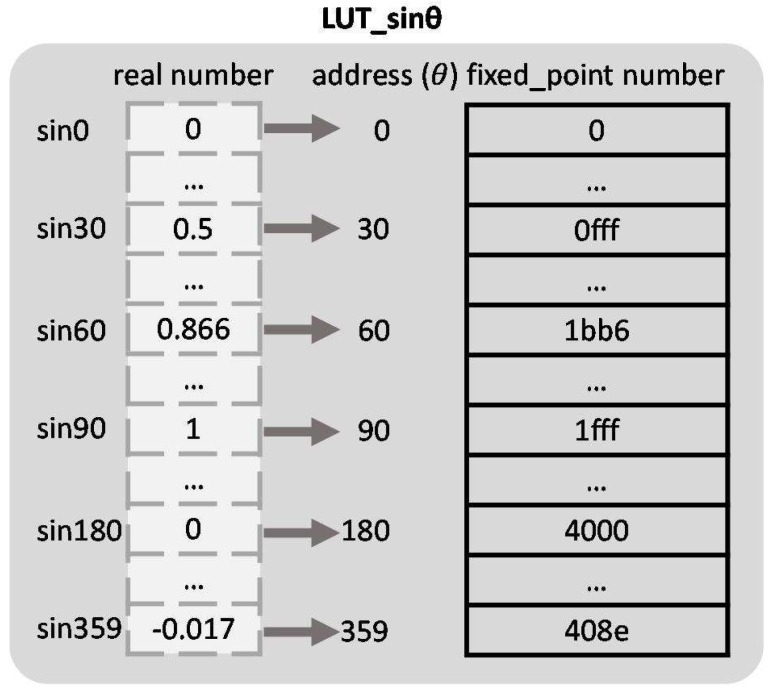
Example of the look-up-table (LUT) for sinθ.

In this paper, we propose a look-up-table (LUT) solution for sinθ and cosθ determination, without any accuracy loss as e.g., in the iterative CORDIC solution. A fixed-point representation is applied, as shown in Figure 1 for the example of the sinθ LUT. These LUTs for sinθ and cosθ replace the time-consuming runtime computation. In the LUTs, the sinθ and cosθ fractional values are scaled by a certain factor, $8192\;(2^{13})$ in Figure 1, and two`s complement notation is used. After computing Equation (1), we truncate the least significant 10 bits of the results to obtain the same number of bits as applied for the *ρ* discretization.

**Figure 2:**
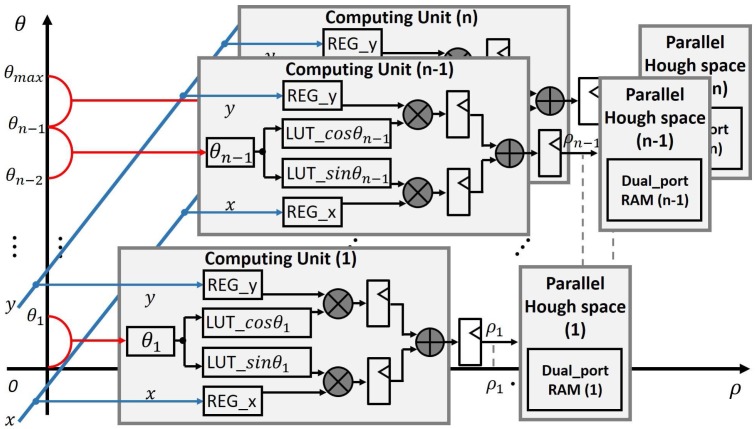
Hardware implementation for *ρ* and *θ* computation with *n*-fold parallelism.

The computing unit for (*ρ*, *θ*) is divided into *n* parallel parts with additional pipeline architecture, as illustrated in Figure 2, where *n* is a fraction of the chosen number of discrete *θ*-values of the Hough space. Each parallel part contains two multipliers, two look-up tables (implemented as RAM) for sin *θ* and cos *θ*, one adder, and pipeline registers between the part-internal computing units. The complete LUTs for sin *θ* and cos *θ* are distributed across the local LUTs of the parallel parts. For example, if $\Delta \theta =1^{\circ }$ and n=10 are chosen, the complete LUTs have 360 entries which are distributed as 36 entries in each local LUT. The image-edge-pixel coordinates (x,y) are the inputs of the module of Figure 2 for parallel computation of *n* corresponding (*ρ*, *θ*) pairs in the Hough space. The parallelisms *n* is kept flexible in the developed architecture by the usage of counters. The total transformation of *p* image-edge pixels into Hough space reduces to $\frac{\theta_{max}}{n\cdot \Delta \theta }\cdot p+\alpha$ clock cycles by the described architecture, where *α* is the pipeline delay. This corresponds to an *n*-fold speed-up in comparison to a conventional architecture.

### 3.2. Parallelized Voting-Procedure Implementation

**Figure 3:**
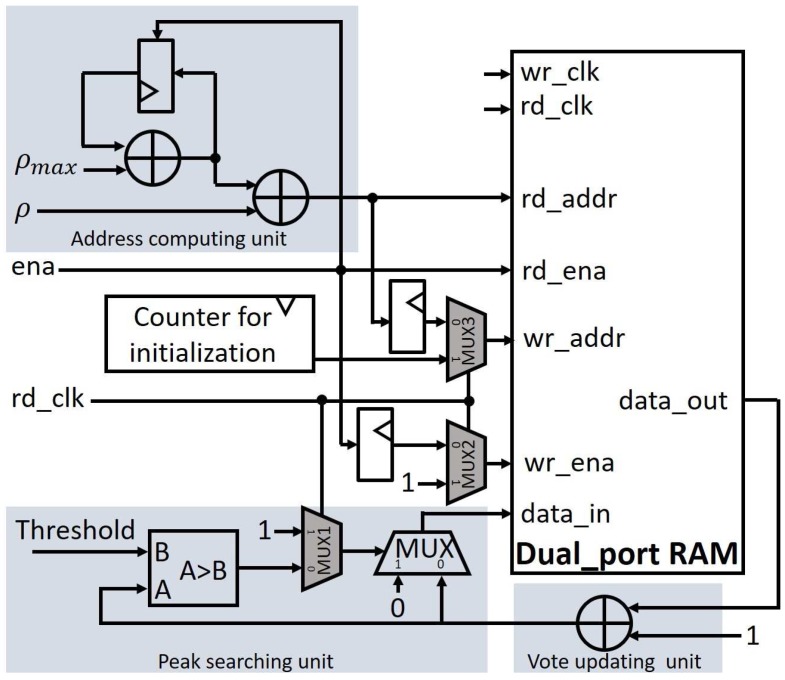
One of the parallel modules for implementing the Hough space by dual-port memory with a pipelined voting procedure.

The Hough space is also divided into *n* parallel modules, which correspond to *n* computing units. Each parallel module is implemented by one dual-port memory block and a few of logic elements as illustrated in Figure 3. A global enable signal (ena) controls the transformation of (*ρ*, *θ*) to the 1-dimensional memory address and the read/write for the dual-port memory. The inputs of the Hough space are the calculated (*ρ*, *θ*) values from the computing units of Figure 2. A register between the address of the read port and write port is inserted to make sure that the new vote value can be accumulated and written back to the read address calculated by the address-computing unit.

**Figure 4:**
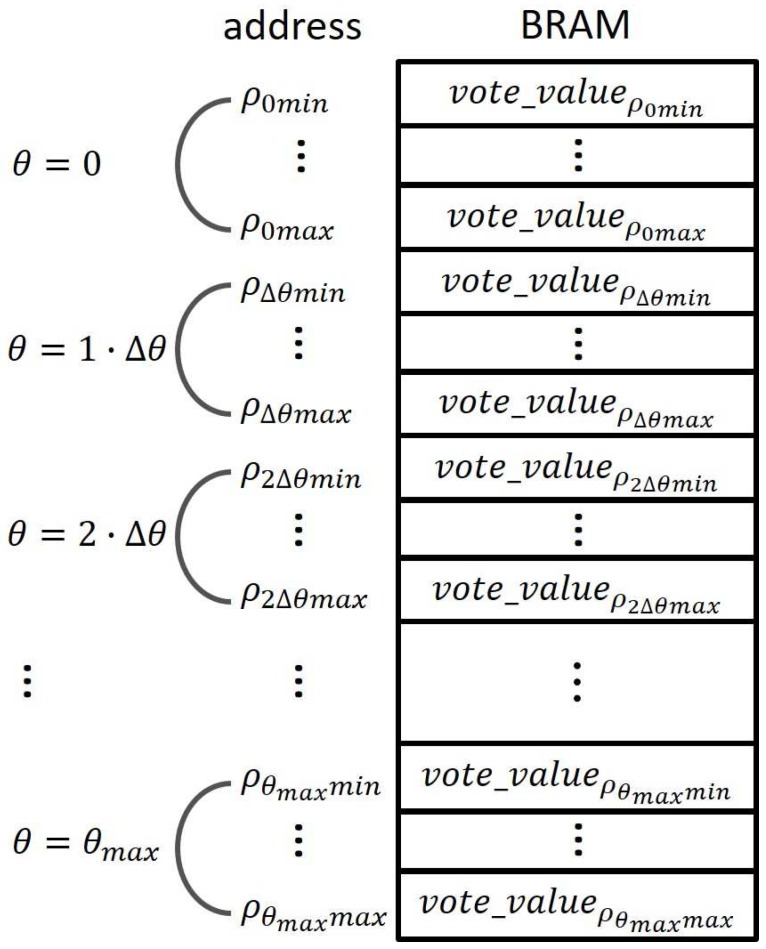
Mapping of 2-dimensional Hough space with (*ρ*, *θ*) into 1-dimensional memories.

To avoid potential read-write conflicts at the same address, the 2-dimensional Hough space with (*ρ*, *θ*) is mapped onto 1-dimensional memory blocks, according to the concept of Figure 4. As a result, the address computing unit in Figure 3 can produce a 1-dimensional memory address in each clock. To implement $\rho +\theta \cdot \rho_{max}$, an adder with feedback input can replace a multiplier since *θ* is progressively increased in each clock. Finally, in every clock one pair of (*ρ*, *θ*) can be attained in each block, there is still no conflict since the *θ* is different in every clock cycle.

Generally, the voting procedure is implemented by increasing the vote value at the corresponding location (*ρ*, *θ*) of the Hough space and compare the vote value with a pre-defined threshold to identify straight lines. For an input image with x×y pixels, $\rho_{max}=\sqrt{x^{2}+y^{2}}$ in units of pixel distances, when the coordinate origin is set as top left image corner. Since a unique representation in polar coordinate is needed for all straight lines through every edge pixel in Cartesian coordinates, *ρ* has to carry a sign to distinguish the position when the angle *θ* is ranging from $0^{\circ }$ to $180^{\circ }$. In this research, the angle *θ* is defined to range from $0^{\circ }$ to $360^{\circ }$, which limits *ρ* to nonnegative numbers. To further decrease storage requirements, the coordinate origin is moved to the geometrical center of the input image, which reduces $\rho_{max}$ by a factor 2. When we use a word length of *B* bits to express the vote value, the total memory for the Hough space needs consequently $\frac{\sqrt{x^{2}+y^{2}}}{2}\cdot \frac{\theta_{max}}{\Delta \theta }\cdot B$ bits.

In the ideal case, a straight line with *n* pixels results in a vote value at the corresponding (*ρ*, *θ*) position of the Hough space, which is equivalent to this pixel number *n*. However, due to the quantization error caused by the choice of Δρ and Δθ, the votes are usually distributed over a small range around this corresponding peak point in the Hough space. The most popular practical way to find the correct polar coordinates of straight lines under these practical limitations is the threshold value method, as it does not require too much computational effort. We have designed a parallel peak-searching unit as shown in Figure 3, which is associated with the voting procedure. The (*ρ*, *θ*) pair for an identified straight line is outputted when the vote value at a Hough-space location becomes larger than a pre-defined threshold. At the same time, the stored vote value at this Hough-space location is reformatted to zero, in preparation of the straight-line detection process for the next image frame.

One of the challenges for the real-time implementation is the initialization of the Hough space, because the Hough space cannot be initialized by one control signal, e.g., a reset signal. The previous research reports in the literature [13,25] mainly optimize the hardware implementation of the Hough space without the discussion of problem of the Hough-space initialization and the complete system structure for video-based applications. The simplest way is to initialize the vote memory for implementing the Hough space after finishing the voting procedure for the current frame. However, this means that the HT for next image frame cannot start until the initialization of the Hough space is finished. As a result, the processing time for one image frame becomes the sum of the actual HT execution time ($\tau_{HT}$) and the Hough-space initialization time ($\tau_{IHS}$). In fact, to judge applicability for real-time processing of a video-frame sequence, the previous works must additionally include the initialization time for the Hough space after each frame in their processing-time estimate.

**Figure 5:**
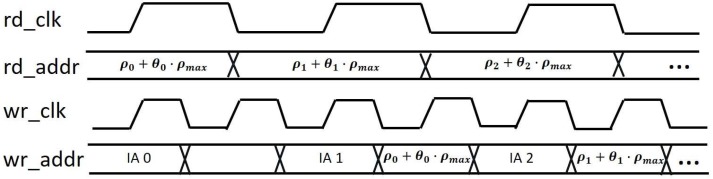
Dual-clock scheme with dual-write processing mode.

In this paper, we implement a parallel initialization method with double clock operation for the write port, which initializes the Hough space during the voting procedure, as shown in Figure 5. Additional multiplexers (MUX1, MUX2, and MUX3) are added to handle the address, write-enable signal and initialization data of the write-port of the dual-port memory in Figure 3, i.e., for switching between vote and initialization mode of the Hough space.

The address counter for the initialization (IA) starts from 0 and increases one by one on the negative edge of the write clock (wr_clk) when the read clock (rd_clk) is high. On the other hand, the register in the address-computing unit latches the next vote address $\rho_{i}+\theta_{i}\cdot \rho_{max}$ on the negative edge of the read clock (rd_clk). Switching between vote and IA mode is also controlled by rd_clk, i.e., the location assigned for IA can be overwritten with 0 when rd_clk is high, while normal voting proceeds when rd_clk is low.

To avoid conflicts between IA and normal voting, we added an additional flag bit to each voting location of the Hough space, which is used to adjust Hough-space updating to the following three cases:

**A**  If the flag bit at the IA address is different from the last bit of the frame number, the stored data is still the voting result for the previous frame. Thus, the vote value at the IA address is initialized to 0 and the flag bit is changed to the last bit of the frame counter, i.e., to the current frame.
**B**  If the flag bit is different from the last bit of the frame number and if a new vote has to be accumulated at the write address, the vote value is initialized to 1 and the flag bit is assigned to the last bit of the frame counter.
**C**  If the flag bit is equal to the last bit of the frame number, this Hough-space location is already initialized for the current frame. Thus, the flag bit is not changed and operation continues according to the vote mode.

In particular, the vote operation has higher priority than the initialization when the vote address calculated by $\rho_{i}+\theta_{i}\cdot \rho_{max}$ is the same to the IA as described in case B. Due to the parallelization of Hough-space initialization and actual HT execution, the computation time for one frame is only limited by the maximum of $\tau_{HT}$ and $\tau_{IHS}$ (max ($\tau_{HT}$, $\tau_{IHS}$)) where $\tau_{HT}$ is usually larger than $\tau_{IHS}$. In other words, the initialization of the Hough space is hidden by execution in the background of the actual HT processing and has normally no effect on the speed performance of the HT in video-sequence processing.

### 3.3. FPGA-Prototype for Straight-Line Detection

**Figure 6:**
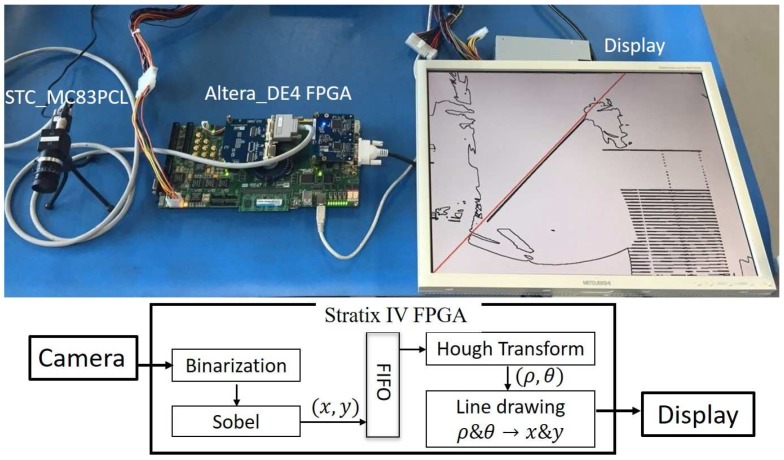
Prototype system for Hough transform (HT)-based straight-line detection.

A prototype system is designed as shown in Figure 6, where a STC_MC83PCL camera captures the video with 1024 × 768 resolution at 30 fps. The system core is implemented on a DE4-230-C2 platform board with a Stratix IV (EP4SGX230KF40C2) Altera FPGA device (Terasic, Taiwan).

**Figure 7:**
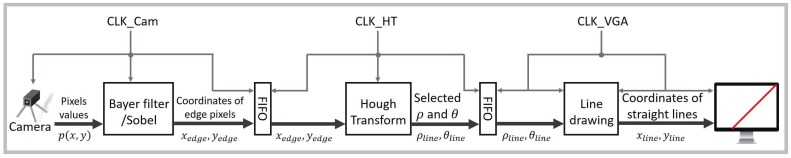
Prototype system for straight-line detection in video inputs.

The system core consists of three major modules for straight-line detection from a video sequence, which are implemented on the FPGA as shown in Figure 7. These modules realize the 3 steps into which straight-line detection can generally be divided. The first module is a preprocessing part for edge detection using the Sobel operator. The main objective of this module is to extract the image edges and finally outputs the coordinates of those edge pixels to the second module, which realizes the actual HT. The computational cost of the HT is mostly dependent on the number of the edge pixels. It means that the edges must be precisely detected from the input image. In order to decrease the effects of the noise, a binarization of the initial Bayer filter results with the thresholding method is performed before the Sobel edge-detector operation. The Cartesian coordinates of the edge pixels obtained by the Sobel operator, with their origin placed at the geometrical center of the input image, are stored in a FIFO for serial input to the second HT-execution module. Finally, the third module transforms the polar coordinates (*ρ*, *θ*) of the detected lines again to the Cartesian-coordinate space for line drawing on an output display.

## 4. Experimental Results

### 4.1. Performance Analysis

In our developed prototype system for straight lines detection, the parallelized Hough space for vote accumulation during the HT consists of 8 parts. Further, the resolution parameters Δρ and Δθ are defined as 1 pixel unit and $2^{\circ }$, respectively. In the case of a video sequence with 1024 × 768 pixels per frame, $\rho_{max}$ is 640 = ($\frac{\sqrt{1024^{2}+768^{2}}}{2}$) as the coordinate origin is set at the geometrical center of the input image. Otherwise, $\rho_{max}$ would be 1280, if the coordinate origin is set at top left corner of each frame. Accordingly, the angle *θ* is defined in range from $0^{\circ }$ to $360^{\circ }$, so as to obtain a nonnegative *ρ*. In summary, the total storage requirement for the Hough space becomes 1,267,200 = ($640\times \frac{360}{2}$× 11) bits where the word length for each vote value is 11-bit.

In addition, the LUT storage needs 5400 = ($\frac{360}{2}\times 15\times 2$) bits. Each word for expressing sin *θ* or cos *θ* is chosen to have 15 bits, where the most significant bit is the sign bit. Furthermore, for computing sin *θ* and cos *θ*, the LUT solution has much more flexibility to adjust the resolution for Δθ in comparison to the previous works [13,25], since any Δθ resolution according to the request of the target application can be initialized in the storage. On the other hand, Δθ is the main factor determining the storage requirements of the Hough space.

As described in Section 3.1, the computation of (*ρ*, *θ*) and the voting procedure for each frame with *p* edge pixels consumes $\frac{\theta_{max}}{n\cdot \Delta \theta }\cdot p+\alpha$ clock cycles. In the case of $\Delta \theta =2^{\circ }$ and parallelized Hough space with n=8, each unit of the distributed LUT storage contains 23 = ($\frac{360}{2\times 8}$) initialized values. Additionally, the pipeline delay *α* becomes 5. Consequently, the proposed architecture can attain $\frac{f_{max}}{\frac{\theta_{max}}{n\cdot \Delta \theta }\cdot p+\alpha }$ fps, where $f_{max}$ is the maximum clock frequency for line-detection-system operation.

The prototype system has three clock domains for camera capturing, line-detection by HT, and straight-line-display operation. The Bayer filter for transforming RGB-color input images to grayscale, the binarization unit for converting grayscale to binary, and the Sobel filter for edge detection adopt the clock signal of the camera with 29.5 MHz pixel frequency. Then, the HT unit uses the synthesized maximum frequency ($f_{max}$) of 200 MHz. Finally, the display timing with XGA video input (1024 × 768 pixels per frame and 30 fps) at 60 Hz requires a 65 MHz pixel frequency.

Due to the pipeline architecture, after the delay of line buffers, the edge-detection module can process each frame in real-time (at 30 fps). Generally, only a small part of pixels (p) in a frame (1024 × 768 pixels) is categorized as edges pixels. Accordingly, the processing time for each edge pixel $\tau_{pixel}$ is $\frac{\theta_{max}}{n\cdot \Delta \theta }+\frac{\alpha }{p}\approx \frac{\theta_{max}}{n\cdot \Delta \theta }$, since *α* is much smaller than edge pixel number *p*. In the case of $f_{max}$ = 200 MHz, $\tau_{pixel}$ is only 115 ns.

**Figure 8:**
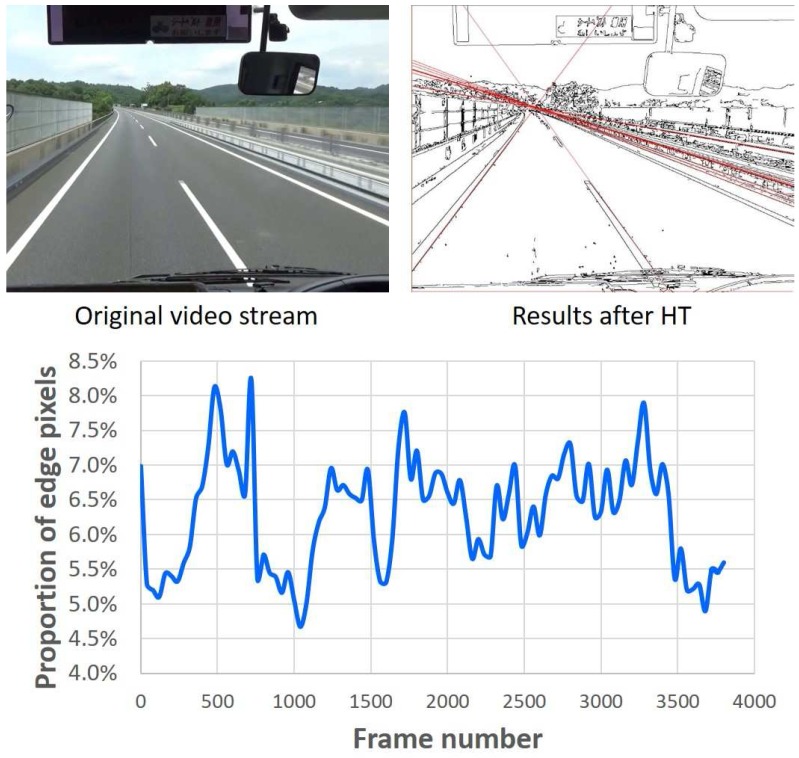
Analysis of the proportion of edge pixels in a highway video.

Lane detection is a very useful technology for avoiding car accidents or reducing the number of human fatalities. We have used 3800 frames from a highway video with a resolution of 1024 × 768 pixels, to analyze the percentage distribution of obtained edge pixels, with results shown in Figure 8. The determined edge-pixel proportion ranges from 4.5% to 8.5% with an average of about 6%. Consequently, the average processing speed of developed prototype system for HT-based straight-line detection is 5.4 ms per frame at 200 MHz working frequency.

The speed performance is compared to the state-of-art previously developed systems [13,25], which are implemented in hardware on specific design platforms. In [13], a Canny edge detector was presented for edge-pixel determination in the processed target video with 1024 × 768 pixels resolution and subsequent sequential processing of those edge pixels. The hardware system in [13] needs 0.24 μs per pixel for edge-pixel classification and 15.59 ms on the average for straight-line determination per frame. In [25], an off-chip memory pre-stores the binary-feature image with run-length encoding for reducing the computing complexity. A block-based processing element computes *ρ* for a specific *θ* with respect to all pixels in the block. A computation time in range of 2.07–3.61 ms with 180 orientations for image resolution of 512 × 512 pixels results, without inclusion of the edge detection procedure. In the same manner, only the edge pixels are processed also in [13]. Nonetheless, the complete binary-feature image has to be traversed in [25] for transformation into the polar space. In particular, the binary-feature extraction with an encoding module, which should be much more complex than the edge detection, is not included in the hardware prototype system of [13].

Due to the different processing mechanisms (i.e., edge pixels or entire image) and the different image resolution, a normalized speed, which illustrate the computation cost for each pixel of the original image frame, is used for a fair comparison. As shown in Table 1, the average speed of this work is much faster than [13], which applies the same HT mechanism for every edge pixel. Furthermore, this work is also superior to [25], even though [25] uses a higher operating frequency of 200 MHz. Additionally, our reported hardware prototype can process even a high-definition video (1920 × 1080 pixels) at 14.3 ms/frame on average, i.e., with more than 60 fps.

### 4.2. Hardware Resource Usage

As mentioned before, the developed prototype system has been implemented on a DE4-230 platform board with Stratix IV EP4SGX230KF40C2 FPGA-device. Apart from the developed architecture for straight-line detection by HT, the developed prototype integrates the pre-processing unit, containing a Bayer filter, a converter for color to gray image, a unit for binarization, a Sobel edge detector, and an edge coordinate FIFO. The additionally implemented post-processing module realizes a line-drawing function for the detected straight lines after back-transformation to Cartesian coordinates from the output results from the HT module.

Table 2 shows the hardware resource usage of each module in the developed prototype system, including the module for clock generation. Specifically, the pre-processing module includes the implementation of a Bayer filter, a converter from RGB to binary image, and a Sobel filter. The HT module contains the straight-line-detection process of the Hough Transform, i.e., computation of all *ρ* and *θ* in the Hough space for each edge pixel and the voting procedure. The line-drawing module implements the back-transformation of the polar HT results to the Cartesian coordinate system, which is used to display the straight lines according to the determined (*ρ*, *θ*) pairs. Finally, the clock part, based on a phase-locked loop (PLL), generates the clock signals for the other modules and the display. In particular, the parallel computing unit of Figure 2 consumes 16 (2 × 8) multipliers in HT module.

The comparison of hardware resource usage for the HT is demonstrated in Table 3. In [13], the angle increment ($\Delta \theta =0.8952^{\circ }$) leads to two times higher resolution than in this work with ($\Delta \theta =2^{\circ }$), but requires almost two times larger memory usage. In [25], the on-chip memory usage could be reduced, because the voting results are only temporarily stored in on-chip memories and then transferred to off-chip memories. In fact, the memory usage of the Hough space should be affected only by the resolution of (Δρ, Δθ). In other words, the previous works in [13,25] and this work should consume the same size of memories when the resolution of (Δρ, Δθ) is the same. On the other hand, the LUT-storage requirements amount to only 0.36% of the total memory usage. In spite of this small hardware consumption, the LUT-solution for sinθ and cosθ enables high flexibility in resolution of the increment of *θ* (Δθ) and high calculation speed.

The comparison of combinational adaptive LUTs (ALUTs) essentially represents the hardware resource efficiency. In contrast to [25], this work and [13] implement an entire HT system for straight-line detection, while Chen et. al. in [25] only implement part of the HT system, without camera input, edge-feature extraction and output module. Particularly, the calculation in [25] has been partly transferred to the not-implemented preprocessing part, so that a hardware implementation with relatively low cost and good performance could be attained. Except for 16 multipliers, the usage of ALUTs in our work is almost the same as in [25]. With respect to the complete system-level comparison, this work consumes only one fifth of the hardware resources required in [13]. In addition, for application in a practical video-based straight-lines detection system, the processing-time expense of the initialization for the Hough space is not but should be included in [13,25]. In other words, without a parallel initialization solution, the real processing time for each frame is expect to be much larger than reported 15.59 ms [13] or 2.07–3.01 ms [25].

## 5. Conclusions

In this paper, instead of the Coordinate Rotational Digital Computer (CORDIC) algorithm, we applied a look-up table (LUT) solution for computing sin *θ* and cos *θ*. Besides the flexible resolution for the increment of *θ* (Δθ), the 2-dimensional Hough space could be transformed to a 1-dimensional array due to the regularity of Δθ. Consequently, we were able to parallelize the Hough space into *n* parts with parallel voting procedure, which enables an FPGA-based hardware implementation allowing real-time line-detection solutions for high-resolution video input data, in spite of the computational complexity. In particular, the developed parallel initialization for the Hough space, hidden by execution in the background of the actual HT processing, additionally contributed to the achievement of video-based straight-line detection with a speed of 5.4 ms frame for XGA (1024 × 768 pixels) videos for the case of architecture implementation on a Stratix IV EP4SGX230KF40C2 FPGA. This demonstrates practical usability in time-critical real-time applications like lane detection in driver-assistance systems for automotive security, which is one of our practical development tasks. An important advantage of our hardware architecture is the possibility of implementation as low power and small size ASICs (application specific integrated circuits) for cases of applications which are power restricted, hardware-size restricted or have high production volumes.

In order to achieve reasonably small memory usage, the resolution parameters Δρ and Δθ of the Hough space are defined as 1 pixel and 2 degrees, respectively, which leads to sufficient accuracy in most practical cases. In our future research, however, we plan to study the influence of Hough-space resolution on accuracy in further detail, to determine the tolerable reduction limits of the resolution in given practical applications. Other future investigation subjects are the reduction possibility for LUT usage by exploiting the relation cos *θ* = sin $\left(\theta +\frac{\pi }{2}\right)$ as well as possible improvements of the simple threshold-value method for finding the potential straight-line candidates during Hough transform as, e.g., reducing multiple line candidates in a local Hough-space surrounding to just the one candidate with the highest vote value. Otherwise, the edge-detection algorithm by Sobel filter with threshold mechanism has also improvement space, e.g., by developing a method for removing the remaining noisy edge-pixels.

## Figures and Tables

**Table 1:** Comparison results for straight-line-detection speeds.

	[13]	[25]	This Work
Working frequency	200 MHz	200 MHz	200 MHz
Image resolution	1024 × 768	512 × 512	1024 × 768
Processing speed (ms/frame)	15.59	2.07–3.61	5.4
Normalized speed (ns/pixel)	19.8	10.8	6.8

**Table 2:** Hardware resource usage of each module in the prototype system for line detection.

	Pre-Processing	HT	Line Drawing	Clock
Logic elements	887	850	788	131
Registers	551	645	166	97
Memory (bit)	51,490	1,513,712	38,848	0
DSP block	24	8	16	0
Total PLLs	0	0	0	1

The Hough space occupies 1,267,200 bits; the LUT storage units for sin *θ* and cos *θ* take 5400 bits; the (*x*, *y*) coordinate FIFO spends 180,224 bits; the memory to store the (*ρ*, *θ*) pairs for line drawing uses 60,888 bits.

**Table 3:** Comparison of hardware-resource usage to previous straight-line detection systems using the Hough Transform.

	[13]	[25]	This Work
Combinational ALUTs	15,704	855	2656 (850)
Registers	13,727	421	1459 (645)
On-chip Memory (bit)	3,052,544	233,360	1,604,050
Off-chip Memory (bit)	0	3,270,032	0
Multiplier	8	0	16
